# Clues to Non-Invasive Implantation Window Monitoring: Isolation and Characterisation of Endometrial Exosomes

**DOI:** 10.3390/cells8080811

**Published:** 2019-08-01

**Authors:** Alice Luddi, Natasa Zarovni, Erika Maltinti, Laura Governini, Vincenzo De Leo, Valentina Cappelli, Luis Quintero, Eugenio Paccagnini, Francesca Loria, Paola Piomboni

**Affiliations:** 1 Department of Molecular and Developmental Medicine, University of Siena, 53100 Siena, Italy; 2 Exosomics; SpA, 53100 Siena, Italy; 3 IMER. Instituto de Medicina Reproductiva, 46009 Valencia, Spain; 4 Department of Life Sciences, University of Siena, 53100 Siena, Italy

**Keywords:** extracellular vesicles, liquid biopsy, implantation window, endometrium, uterine flushings, cervical brush

## Abstract

Despite the significant advances in the last decades, low implantation rate per transferred embryo still remains a major concern in assisted reproductive techniques, highlighting a need to better characterize endometrial receptivity also by mean of specific biomarkers. Based on physiology and on the intimate contact with endometrium as the tissue of interest, in this study we developed and validated an optimized protocol that uses extracellular vesicles (EVs) recovered from uterine flushings and from a cervical brush, the latter never used until now as an EVs source, as surrogates for endometrial biopsies. This method combines the safety of sampling with the ability to study the expression profile across the uterine cycle. We have compared the yield and composition of EVs recovered from different biofluids samples and fractions thereof, opting for chemical precipitation as the EV isolation procedure, assuring the highest yield without introducing any bias in specific EV recovery. Moreover, collected EVs, in particular exosome-like vesicles, express putative endometrial markers, such as glycodelin A and receptors for estrogen and progesterone, thus confirming their endometrial origin. We also identified uterine flushing EVs, in particular those recovered from its mucous fraction, as the richest source of endometrial transcripts, likely correlated to cellular (epithelial) origin of these vesicles. Finally, our pilot quantitative assessment of three endometrial gene profiles, in samples collected at different time points along the luteal phase, revealed the fluctuations apparently recapitulating gene expression variability prior reported during the menstrual cycle. Unlike tissue biopsy that is subjected to inter- and intra-sample differences, our data suggest that EVs from liquid biopsies (from uterine flushings and a cervical brush) obtained through less-invasive procedures, can be substrate to detect and track the tissue representative expression profiles, better depicting the total endometrium complexity.

## 1. Introduction

Notwithstanding the significant progresses achieved in the last decade, low implantation rate per transferred embryo, likely stemming from a suboptimal uterine receptivity, still remains a major problem in assisted reproductive techniques (ART) [1]. Due to the poor predictive value of standard morphologic and hormonal parameters, alternative biomarkers of endometrial receptivity are actively investigated [2,3,4]. Recently, specific endometrial tissue trascriptomic fingerprinting has been depicted, enabling the classification of receptive or not receptive endometrium [5]. The translation of tissue biopsy based assays into the clinical routine is hampered by their complexity, costs, and invasive nature, as well as their limited capacity to capture the heterogeneity of the tissue of interest [5]. Hence, the ability to tailor the timing of embryo transfer in order to raise take-home baby rate remains one of central clinical needs in reproductive medicine today. Successful embryo-implantation is only possible for a short period of time when the hostile uterine lining transforms to a hospitable surface to accept the embryo. The endometrial receptivity is a dynamic morphologic and molecular process that lead the endometrial cells to establish a two-way cross-talk with the embryo [6,7]. Several factors, secreted by the endometrium into uterine fluid, control implantation by either directly affecting blastocyst development and/or by modulating the expression of key adhesion molecules. Some of these are sorted from endosomal compartments into secretory exosomes/EVs and are thus delivered to target tissues in a paracrine (blastocyst) and autocrine manner (endometrium itself) in a selective and specific manner. According to their size and biogenesis, EVs are broadly classified into: (i) exosomes (EXs), 30 to 100 nm in size, originated from endosomal compartment of the cell; (ii) microvesicles (MVs), ranging from 100 to 1000 nm, released from budding and fission of the plasma membrane. To avoid any confusion in EV nomenclature, novel ISEV accepted guidelines recommends the use of using generic term “EV” unless the subcellular origin of the vesicles is undoubtedly demonstrated [8]. EVs are considered as appealing enabling tool for non-invasive diagnostic applications and biomarkers discovery [9,10]; their emerging role in regulation of gametogenesis, fertilization, embryo implantation, and development, make EVs ideal candidate biomarkers for reproductive outcome, and potential targets for therapeutic interventions [11,12]. Accordingly, EXs-derived miRNAs isolated in vitro from endometrial cells have been shown to be critical for biological pathways highly relevant for embryo implantation [13]. It has been reported that EVs released from endometrial epithelium into the uterine cavity contain proteins and RNAs that are related to implantation [14]. As a consequence, recovery and analysis of endometrium-derived exosomes might represent a suitable alternative to invasive uterine biopsies, but the protocols for their specific recovery from uterine flushes and, in particular, the correlation of composition of EVs with cyclic changes in healthy or pathologic endometrium is poorly studied. In this study we set up a robust protocol for EVs isolation from different surrogate biofluids: the uterine flushes (composed of mucus and liquid uterine phases) and the cervical brush, a substrate never used until now. These two sources are evaluated as alternative to other classical liquid biopsy substrates such as serum or urine, that pose the challenges in terms of, respectively, either high complexity or low and intrinsically variable EV composition, likely enabling scarce recovery of endometrium specific EV shuttled markers.

## 2. Materials and Methods

### 2.1. Patients Enrollment and Monitoring

Uterine flushings (UF), cervical cyto-brushes (B), serum (S), and urine (U) used in this study were collected at the Centre of Couple Sterility, Gynecology and Obstetrics Unit, Siena University Hospital (Italy) and at the IMER Clinique, Valencia, Spain. Samples were obtained from women volunteers with proven fertility (*n* = 16). The uterine flushing was scheduled 3, 5, 7, or 9 days after the LH surge (LH + 3, LH + 5, LH + 7 and LH + 9 respectively). The ovulation timing was characterised by measuring the LH surge with specific kit (One Step LH Ovulation Test, AIDE Diagnostic, Shandong, China), confirmed by blood sample evaluation and ultrasound ecographic examination. The uterine flushing was performed by using a disposable catheter for histerosalpingography, filled with 4 mL of physiologic solution, according to published protocols [15]. The sample was immediately recovered from the catheter and centrifuged at 1200 r.c.f. for 10 min in order to separate the mucus (M-UF) from the liquid fraction (L-UF). Each fraction was split into two aliquots; one was stored at −20 °C and the other was stored at room temperature with a chemical preservative (Norgen Biotek, Canada) for 30 days. The overall amount of thus recovered flushing sample varied from 2.5 to 3.5 mL. A cervical brush was obtained by rotating the cytobrus two full turns into the endocervix; after that, the brush heads were deposited into a vial containing 1 mL PBS. Serum was obtained upon blood draw, aliquoted, and stored as indicated (either at RT or frozen). Starting volume of serum used for EV isolation and RNA extraction was 1 mL. Urine was collected in sterile containers and the sediment was removed by immediate 600g/10 min/RT spinning in the bench microfuge. Urine was stored frozen prior to analysis and EV isolation. The urine was thawed and warmed up to 37 °C. Five mL of urine was used to isolate EVs.

The present study was approved by the local Ethic Committee CEAVSE (IRB 20062016), and the written informed consent was obtained from all participants prior to their enrollment. All research was performed in accordance with the Helsinki Declaration and relevant guidelines and regulations.

### 2.2. Treatment of Human Uterine Flushing, Uterine Mucus and Cervical Cytobrush for EV Purification

EVs recovery from L-UF, M-UF and B was performed as described below. Once thawed, all samples were supplemented with protease inhibitors and purified and/or analyzed immediately. M-UF and B were initially digested with the antioxidant and mucolytic agent *N*-acetyl-l-cysteine (20 mg/mL in PBS; Sigma-Aldrich, Saint Louis, MO, USA), followed by incubation for 45 min at 37 °C. After centrifugation at 1200 rpm. for 10 min at room temperature (RT), supernatants were recovered, whereas pellets were washed with phosphate buffered saline (PBS; 1X) and subjected to a centrifugation at 1200 rpm for 10 min at RT. The latter supernatants were added to those previously collected and new obtained pellets were further digested with the enzymatic agent Liberase TL/TM (0.5 WU/mL in PBS; Roche Diagnostics, Mannheim, Germany), followed by incubation for 5 min at 37 °C. To remove cells and cell debris, M-UF and B-derived samples as well as L-UF were subjected to centrifugation at 1200 rpm for 20 min at RT (in the text this low-speed bench centrifugation is often referred to as a pre-clearing). Supernatants derived from M-UF and B were added to those previously recovered. All supernatants were supplemented with protease inhibitor cocktail (1:500 working dilution; Sigma-Aldrich), followed by collection and storage at −20 °C of an aliquot of each for further analysis of vesicular content.

### 2.3. EV Purification

EV purification from biological samples was performed by either peptide-mediated affinity (PA), immunoaffinity (IA), or chemical precipitation (CP). PA-based EV isolation was performed using METM kit (NEP, MA, US) according to the manufacturer’s instructions and as previously published [16]. Briefly, samples are prepared and precleared (by 12,000 rpm centrifugation) as described above, divided into 3 aliquots and each brought to a 1 mL volume with PBS. Samples were incubated with the ME^TM^ reagent and mixed for 15 min at RT with rotation. The incubated samples were centrifuged at 10,000× *g* for 7 min at RT using a bench-top microcentrifuge. All samples were washed three times with PBS and final pellet was resuspended in appropriate kit comprised buffer. EV recovery by IA was based on the use of commercially available NH2-latex beads (400 nm) (HansaBioMed Life Sciences, Tallin, Estonia) coated with anti-CD9 and anti-CD63, following the manufacturer’s instructions and prior published protocols [16]. Briefly, each sample was incubated with appropriate beads overnight at 4 °C on a rotator. Upon exosome binding, beads were recovered by top bench centrifugation (5000× *g* for 10 min) and washed three times with PBS 0.05% Tween-20 (PBST) prior to RNA extraction. For EV isolation by CP, samples were treated with Exo-Prep precipitation reagent (HansaBioMed OU, Tallin, Estonia), according to the manufacturer’s instructions. Briefly, upon addition of the Exo.Prep reagent, the samples were incubated for 1 h in ice. The exosome pellet was recovered by the bench centrifugation at 7000× *g*, for 10 min, and washed three times in PBS prior to RNA extraction.

### 2.4. RNase A Protection Assay

To degrade unprotected RNA and ascertain the isolation of RNA only confined within EVs, purified EV pellets were treated with RNase A (0.1/0.5 mg/mL; Thermo Fisher Scientific) for 20 min at 37 °C, followed by enzyme inactivation. Untreated purified EVs were tested in parallel as controls.

### 2.5. Real-Time RTqPCR

EV-associated RNA was isolated from each purified EV pellet using an RNA Isolation Kit, according to the manufacturer’s protocol (HansaBiomed OU). Aliquots L-UF-, M-UF, and B- derived RNA samples were used for assessment of RNA concentration and integrity by Qubit^®^ RNA HS Assay Kit (Thermo Fisher, Waltham, MA, USA) and the Agilent 2100 Bioanalyzer instrument using the Small RNA kit (Agilent Technologies, Santa Clara, CA, USA), respectively. To evaluate the expression of Estrogen Receptor-1 (ESR1), Progesteron Receptor (PGR), Progestagen Associated Endometrial Protein (PAEP), and Glyceraldehyde 3-phosphate dehydrogenase (GAPDH) mRNAs, and RNY4 non-coding RNA, Real-Time RTqPCR was performed in triplicate using the iTaqTM Universal SYBR Green One-Step kit (Bio-Rad, Hercules, CA, USA). Forward and reverse primer pair sequences for each gene were (5′–3′): GGCTACATCATCTCGGTTCC and TCAGGGTGCTFFACAGAAA for ESR1; CCTTACCTGTGGGAGCTGTA and GCAGTCATTTCTTCCAGCAC for PGR; ATGGACATCCCCCAG and ACCTTCTTCTCAACA for PAEP; CAATGACCCCTTCATTGACC and TTGATTTTGGAGGGATCTCG for GAPDH; GTCCGATGGTAGTGGGTTA and AAAGCCAGTAAATTTAGC for RNY4. All PCR reactions were run in CFX96TM Real-Time PCR Detection System (Bio-Rad, Hercules, CA, USA) using 96-well reaction microplates (Bio-Rad, Hercules, CA, USA), and analyzed by CFX Manager SoftwareTM to calculate threshold cycles (C_t_).

To appreciate variations in RNA levels results are expressed using a comparative C_t_ method. In particular, Ct values for common exosomal RNAs such as RNy4 and GAPDH are converted into quantities in a linear scale (assuming amplification efficiency of 100% for each mRNA, (2^(40 − Ct)^). GAPDH and RNY4 mRNA were used as reference genes to normalize the values for endometrial specific genes, namely PAEP, ESR1 and PGR using the δC_t_ and/or δδC_t_ method. For the latter RNA extracted from endometrial primary cell cultures was used as a reference sample [17].

### 2.6. Protein Quantification

Measurements of protein concentration in all analyzed samples were performed using the Pierce™ BCA Protein Assay (Thermo Fisher Scientific), according to the manufacturer’s instructions.

### 2.7. Nanoparticle Tracking Analysis (NTA)

Nanoparticle tracking analysis (NTA) was performed using a NanoSight LM10-HS microscope (NanoSight, Ltd., Amesbury, UK) equipped with a 405 nm laser. Samples were diluted with PBS to reach a working concentration of 20–120 nanoparticles per frame and/or 107–109 nanoparticles/mL. For each sample, three 30-sec videos with a frame rate of 30 frames per second were recorded. Temperature was monitored and recorded throughout the measurements. Captured videos were analyzed by NTA software (version 3.2) (Malvern Panalytical, Herrenberg, Germany) to determine nanoparticle concentration and size with relative standard error. For analysis, automatic settings were used for blur, minimum track length, and minimum expected particle size. Prior to analysis, calibration of the NanoSight system was performed using polystyrene latex microbeads with a size of 100, 200 nm, and 400 nm (NanoSight, Ltd.).

### 2.8. ELISA-Based Exosome Immunocapture and Quantification

For EV quantification by detection of surface biomarkers, exosome-based sandwich ELISA assay (ExoTESTTM; HansaBiomed Life Sciences) was performed in 96-well microplates, according to the manufacturer’s protocol. Briefly, microplates coated with primary capture antibodies anti-CD9 and anti-CD63 were incubated with either standards and samples overnight at 4 °C. Microplate wells were treated with primary detection antibodies anti-CD9-bio, followed by incubation with HRP-Streptavidin-conjugated secondary antibodies and TMB Substrate Solution. Stop Solution (2N H2SO4) was added to each microplate well. Assay was calibrated using a standard curve prepared with linear dilutions of exosomes purified from human plasma (HBM-PEP; HansaBioMed Life Sciences) embedded in the ELISA kit as standards. Optical densities were determined using a microplate reader Infinite_M1000 (TECAN) set at 450 nm.

### 2.9. TEM Analysis

Samples were prepared for transmission electron microscopy (TEM) by the conventional negative staining procedure performed on. 5 different samples. In brief, 5 µL aliquots of EV suspension were sedimented for 2 min onto a 300 mesh, copper/carbon-coated grid and then negatively stained with 1% uranyl acetate and observed with a TEM Fei Tecnai G2 spirit at 80 Kv. For the immunogold procedure, fresh EVs were allowed to adhere to 300 mesh nickel formvar/carbon-coated grids, then exposed for 1h to the primary antibodies anti-CD9 (11-354-C100) and anti-CD63 (1B-343-C100), (both from ExBio, Prague, Czech Republic) and anti-flotilline (sc-74566, Santa Cruz Biotechnology, US), diluted 1:150 in PBS. The grids were then washed twice and finally incubated for 1h with the 10 nm gold-labelled secondary antibody (diluted 1:100 in PBS) (British Biocell, Cardiff, UK). After washing to eliminate any non-specific binding of secondary antibody, the samples were fixed using 1% glutaraldehyde, contrasted with 1% uranyl acetate, and observed with a TEM Fei Tecnai G2 Spirit. The obtained digital images were processed with Image J software acquisition.

### 2.10. Statistical Analysis

Statistical analysis has been performed by using Prism v5 (GraphPad Software, La Jolla, CA). Briefly, we applied two-sided paired Student’s *t*-test or Welch’s *t*-test and ANOVA test when appropriate, by performing multivariate analysis and including the correlation coefficient determination and logistic regression. Details are provided in legends for each data figure.

## 3. Results

### 3.1. Optimization of EV Isolation Procedure from 3 Different Biofluids

Two fractions of uterine flushing, the mucous and the liquid one (M-UF and L-UF respectively) as well as cervical brush derived material (B) were processed according to newly optimized protocol including a preclearing and a purification step in order to isolate EV pellets (as detailed in the Material and Methods section). To increase recovery of vesicles potentially entrapped in mucous material from M-UF and B, the step-wise mucolytic and enzymatic digestion was employed. Three isolation methods have been considered based on chemical precipitation (CP), peptide-mediated affinity (PA), and immunoaffinity (IA), chosen as suitable for efficient single step recovery of EVs from small volumes of complex biofluids (see Graphical abstract). The overall comparative evaluation of isolation protocols and sample types from healthy volunteers used for a set-up is shown in the Supplementary Figure S1.

CP has been used as a preferential method in this study as it enabled major total EV RNA yield in preliminary experiments and did not introduce any bias in specific EVs recovery and thus it is suitable for biomarker discovery and characterization phase. However, both PA and IA that are conventionally used as methods of choice for plasma and serum, as well as for spot urine samples [18,19], have confirmed to be feasible methods for working with sample types of interests in this study, thus paving the way for further development of methods for specific isolation and enrichment of desired EV populations.

**Figure 1:**
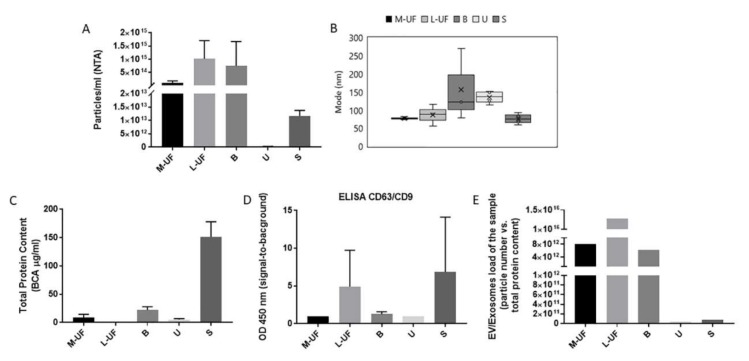
Identification, counting, and quantitative analysis of extracellular vesicles (EVs)/exosomes isolated from different biological fluids at LH+3. Chemical Precipitation has been used as a preferential method in this study. Nanoparticle tracking analysis (NTA) analysis shows particle concentration (**A**) and size (**B**) of EVs from different biofluids (M-UF: mucus fraction of the uterine flushing; L-UF: liquid fraction of the uterine flushing; B: cervical brush; U: urine; S: serum). Total protein content was assessed by BCA assay (**C**), while ELISA was applied to measure the expression of CD63/CD9+ vesicles (**D**). The index of EVs/Exosomes contribution to total sample composition, calculated as a ratio between exosome-sized particle number vs. overall protein content of the sample, is showed in panel (**E**). The data analysis is done using a non-parametric test for multi-group comparison (three replicates each) embedded in a Prism Graphpad software; We used 1-WAY ANOVA followed by Tukey post-hoc test for analysis of particle concentration and ELISA data; All *p*-values < 0.05 are marked with *, < 0.01 with **. To analyse size distribution data, we used an alternative to ANOVA test, Kruskal-Wallis, due to a non-normal distribution observed in some groups; resulting p value>0,05.

The overall content of EVs-exosomes across different biofluids was estimated in samples obtained from healthy donors in LH+3 phase; this time point is selected as determining the start of the midluteal phase of the cycle that conventionally precedes the opening of implantation window [20]. We have first evaluated a total protein content of raw biofluid samples, by assessment of general EV parameters including total EV concentration and size distribution as well as specific EV protein amounts (Figure 1).

Nanoparticle Tracking Analysis (NTA) permitted to obtain size distributions and total concentration of EV-like particles, demonstrating that all samples after processing (where needed) and preclearing (in order to eliminate large debris) contained a consistent number (Figure 1A) of vesicles within the expected size range (80–120 nm) (Figure 1B). In the B sample, a heterogeneous smear of particles sizing up to about 300nm was also observed. The B sample apparently has a high protein content (second only to serum) and high particle number evidenced by NTA, while respectively very few CD9^+^CD63^+^ vesicles were detected in a specific ELISA sandwich assay (Figure 1D). High particle concentration measured in B samples doesn’t correlate well with ELISA measurements. On the other hand, B samples have overall very high protein load. These two observations indicate the presence of contaminant components in B that can cause artifacts still detectable by NTA (i.e., lipid particles, lipoproteins) that can confound the EV counting. In this setting, we consider our ELISA test, designed to recognize CD9 and CD63 co-expressing vesicles, a more specific tool to estimate true vesicle content in the sample than NTA counting. Conversely, M-UF although having a higher total protein load (*p* < 0.05), is relatively poor in vesicles. In line with prior observations, urine and serum also present a very low relative vesicles content (Figure 1A, E) meaning that vesicles and related cargo account for only a very small fraction of a proteome of these complex biosamples. As shown in Figure 1C, despite a very low overall protein content, L-UF samples appears very rich in particles as measured by NTA (*p* < 0.05) (Figure 1A) and ELISA (Figure 1D). This drives us to a conclusion that exosomes largely contribute to a proteome of L-UF, while are less concentrated in its mucous fraction.

**Figure 2:**
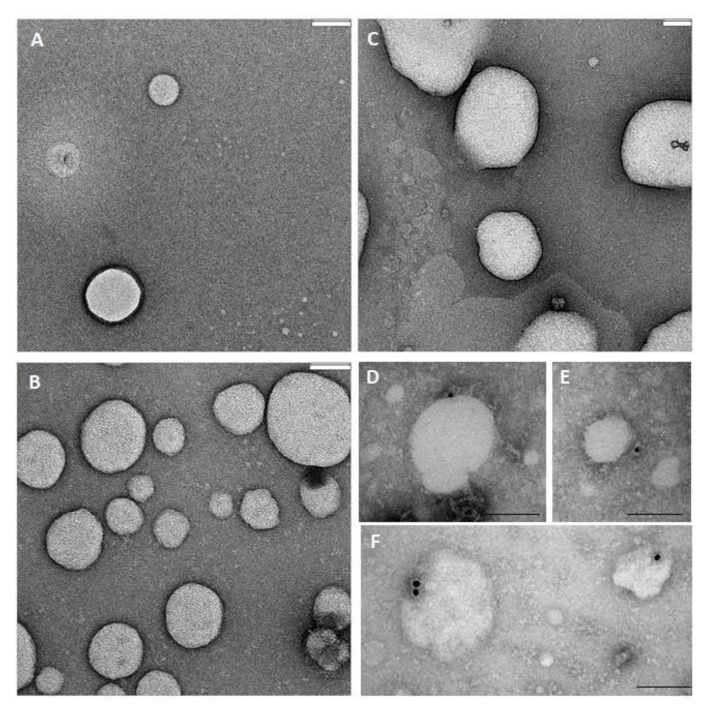
Ultrastructural characterization of Evs/Exosomes from M-UF, L-UF, and B. (**A**–**C**) Representative electron-micrographs of EVs/Exosomes visualized after negative staining, prepared from M-UF (**A**), L-UF (**B**) and B (**C**). Bar = 80 nm. (**D**–**F**) Representative electron-micrograph of immunogold staining in EVs/exosomes prepared from M-UF (**D**), L-UF (**E**) and B (**F**), after immunolocalization of specific markers CD9, CD63 and Flotilin1. Bar= 100 nm.

By confronting the particle number and the total protein content we can appreciate that index of EV contribution to total sample composition in L-UF is 10^4^ higher with respect to both M-UF and B (Figure 1E). This is even more striking when we confront the ratio of total proteins (measured by BCA) and exosomal CD9 levels (measured in ELISA). The size and morphology of EVs were in parallel addressed also by TEM analysis. First images obtained via negative staining (Figure 2A) show that observed particles had expected EV-like morphology and size, in line with NTA profile (Figure 1B). Immunogold further confirmed the identity of EV-like structure by detection of common EV markers like tetraspanins CD9 and CD63, and Flotilin1 (Figure 2B).

As expected, only a portion of vesicles, roughly 20–25%, is positive for each of the common EV markers evidenced by used antibody. The population of EVs released even from a single cell type is very heterogeneous at the molecular level, leading to a detection of “subpopulations” expressing distinct EV markers that sometimes overlap. This heterogeneity is exacerbated in complex biofluids.

We have addressed the possibility that the vesicle composition of UF and B samples might change along the proceeding of the menstrual cycle, by comparing EV counts, size, and protein content in samples obtained from independent donors at different time points, from LH + 3 to LH + 9 (Supplementary Figure S2A–C). Due to ethical restrictions it was not possible to obtain longitudinal samples from the same donors. However, while the size of vesicles observed in UF fractions and the B sample remained unaltered, we confirm the same trend in relative abundance of vesicles in L-UF vs M-UF, with an apparent relative increase of vesicles in B samples in later cycle phases.

### 3.2. EVs/Exosomes Stability

We have also performed the assessment of stability of UF, serum and urine for subsequent EV analysis upon storage at −20 °C or at room temperature (RT, 18–23 °C) for 30 days upon addition of a sample preservative. The latter provides friendly-to-store and ship sample conditions suitable for both research and clinical routine. Common feature of all sample types is excellent preservation of EVs during up to 1 month-storage at RT with respect to −20 °C (Supplementary Table S1) as confirmed by both NTA measurements and ELISA. The number of vesicles appears better maintained at RT.

### 3.3. Analysis of EV RNA

**Figure 3:**
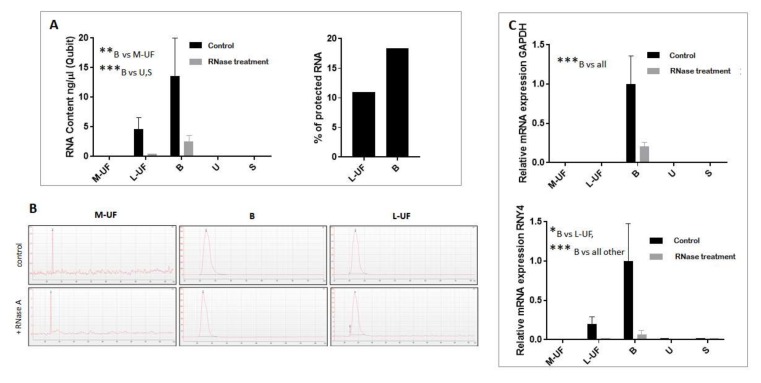
Quantification of t otal RNA extracted from EVs purified from M-UF, L-UF, B, U, and S. (**A**) Total RNA content quantification by direct Qubit^®^ HS assay, with or without RNase treatment. (**B**) Bioanalyzer analysis of total EVs RNA from M-UF, B, and L-UF with or without RNase treatment. (**C**) RT-qPCR amplification of RNAs described to be contained in EVs, namely *GAPDH* and *RNY4*, with or without RNase treatment. We show the average relative expression of *RNY4* and *GAPDH* obtained from three biological replicates per every sample type. 1-way ANOVA and Bonferroni post-hoc test were used to determine statistical significance of observed differences * indicates *p*-value < 0.05, ** *p*-value < 0.01 and *** *p*-value < 0.001.

Total RNA extracted from EVs purified from L-UF, M-UF, and B samples, as well as from available serum and urine from the same donors, was quantified by direct Qubit^®^ kit measurement as well as with RT-qPCR amplification of RNAs described to be contained in diverse vesicle types, namely *GAPDH* and *RNY4* (Figure 3).

*GAPDH* is ubiquitously expressed mRNA that was often used as a housekeeping gene in assessment of EV RNA profiles, although it is not highly expressed in vesicles [21,22]. Y-RNAs, *RNY4* in particular, have been found to be enriched in exosomes [23]. For this reason, this small regulatory RNA was used in this study to estimate and normalize for overall RNA vesicular content. Both absolute total RNA measurement and RT-PCR evidenced high RNA content of EVs purified from B and L-UF while the levels of RNA extracted from M-UF, as well as from serum and urine samples, resulted poorly and therefore beyond the sensitivity limit of the Qubit assay (Figure 3A, left panel). RNase protection assay shows the presence of RNA that is protected from enzymatic digestion in vesicles isolated from L-UF and B (Figure 3A, right panel). The loss of RNA observed upon RNAse treatment of the isolated EV pellets is either due to extravesicular co-precipitated material, or compromised integrity of isolated vesicles during the sample processing. EV encapsulated RNA holds the advantage of the stability and traceable tissue origin. Notably, mRNAs comprise only a minor fraction of a bundle of EV-shuttled RNA species [24]. Byoanalyser assay confirmed a fair recovery of RNA from B and L-UF samples, with the typical profile reported for EV RNA, enriched in a small RNA portion [25]; this profile is not affected by RNase-A treatment (Figure 3B). Likewise, extracted EV RNA also allows for a significant amplification of housekeeping transcripts, in particular *RNY4 (p < 0.001)* (Figure 3C).

*RNY4* and *GAPDH* expression in EVs obtained from L-UF and B was subsequently compared across the luteal phase. As shown in Supplementary Figure S2D,E, both *RNY4* and *GAPDH* are poorly or not detected in M-UF while detected in both L-UF and B, with relative expression across different sample types in line with relative EV concentration as measured by ELISA and NTA.

### 3.4. Expression of Endometrial-Specific Genes in UF and B Extracellular Vesicles

The driving goal of the study was to assess the suitability of EVs from investigated biofluids as surrogates for endometrial biopsies. To trace the portion of endometrial EVs in total EV pellets recovered from each sample type we have addressed the expression of mRNAs known to be specifically expressed in the endometrium and thus used as an indicator of endometrial origin.

**Figure 4:**
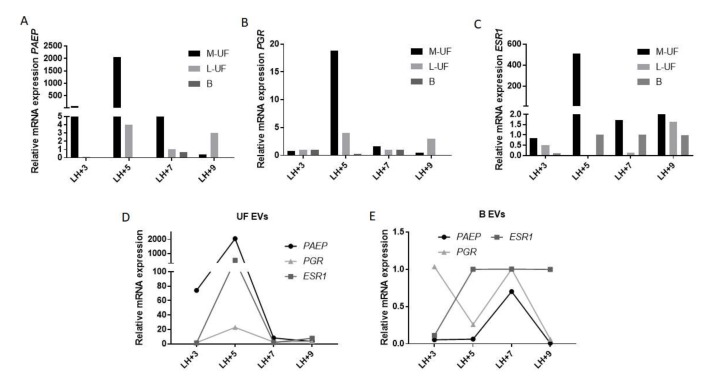
Relative expression of Progestagen Associated Endometrial Protein (PAEP), Progesteron Receptor (PGR) and Estrogen Receptor-1 (ESR1) in UF and B across the cycle phases. EVs/Exosomes associated expression of three putative endometrial transcripts, namely *PAEP* mRNA (**A**), as well as mRNAs for receptors for estrogen and progesterone, *ESR1* (**B**) and *PGR* (**C**), assessed by RT-qPCR. (**D**,**E**) Comparison of the level of expression of endometrial genes *PAEP, PGR*, and *ESR1* in EVs isolated from UF (**D**) and B (**E**) harvested from independent donors at different time points along the luteal phase of the menstrual cycle. The normalization of expression of genes of interest was done using GAPFH and RNY4 as reference genes and the EV-RNA from human endometrial stromal cell cultures as a reference sample.

As shown in Figure 4, consistent expression of all three putative endometrial markers, namely *PAEP* (the gene encoding for glycodelin, GdA), as well as of receptors for progesterone and estrogen, *PGR* and *ESR1* has been detected in vesicles recovered from uterine flushings. This is likely due to the fact that these samples get most intimately in contact with the endometrium lining. Unexpectedly, the mucous fraction (M-UF), though shown to have a low EV RNA burden, gave the highest relative signal for endometrial genes, in particular for *PAEP* that is its most specific and unique tissue trait.

All three genes reach the maximum expression in EVs harvested at the beginning of mid-luteal phase, at day LH + 5 (Figure 4A–C). Also the shift from mucous to liquid UF phase at latest time points is a common observation for all three endometrial genes. Though multifold lower, the expression has been well revealed also in B sample, providing the proof of presence of material, likely vesicular, of potential endometrial origin in cervical secretions.

Overall, we can appreciate that the genes of interest are differently expressed in different sample types and even different sample fractions (mucus vs liquid phase), with an overall high expression in total UF, while they are still detected in B vesicles sample, though with a much lower level and a different temporal pattern of expression (Figure 4D,E).

## 4. Discussion

In this manuscript we tackle the possibility of using EVs recovered from uterine flushings and from a cervical brush, as surrogates for endometrial biopsies. To this purpose we have set up a robust and reliable protocol for sample processing and EVs isolation, with subsequent multiparameter and quantitative characterisation of isolated vesicles. Identification of non-invasive and affordable methods for real time monitoring of endometrium condition and activity, and identification of reliably measurable molecular or biochemical markers, is essential for advancing the field of reproductive medicine. Prior studies have described EVs in uterine fluid, confirming their morphological and biochemical features [11,13,26,27]. The method and timing for uterine cavity flushings and the subsequent uterine fluid sample processing differ across the studies performed in different centers, likely to influence overall yield and quality of the vesicles. Here we propose a protocol for UF harvesting while addressing also the differential composition of UF, typically comprising a liquid and mucous component. The mucous component is usually neglected as a source of EVs. Indeed, this component, that also in our first analysis results poorly in vesicles and their associated RNAs, and likely contains diverse proteinaceous non-vesicular “contaminants”, risks being discarded in the early steps of sample processing. We instead examined the possibility that EVs are differentially distributed in these two phases, and that this might be influenced by the timing of harvesting along the normal menstrual cycle. The mucous component was ultimately enriched in EV-associated endometrial transcripts, in line with their epithelial cells-origin, bringing us to decide to consider and include both components of UF in our analytical practice.

To date, studies employing UF derived vesicles reported the use of an ultracentrifugation-based isolation method. This method, although still widely maintained as a gold standard in fundamental research setting, is not compliant with the actual use of EVs in diagnostic practice. Plenty of data on different sample matrixes shows that methods other than ultracentrifugation have advantages when applied to reduced biofluid volumes, and in particular when used for viscous and complex biofluid sources of EVs [16]. In this study, obtained UF sample volume is typically 1.5–2 mL and the viscosity of the sample can differ across the donors or/and the cycle phases. Theoretically ultracentrifugation-based protocol should be adjusted to viscosity and volume of the sample, therefore being hard to optimise for UF. Hence, we have chosen the EV isolation methods that are not only suitable for small and variable consistency samples, but also display some advantages that rank them highly from the perspective of translational and clinical research: they are quick, efficient, single-step, and cheap, with no need for capital equipment. We have considered three commercially available methods based on polymer-based (chemical) precipitation, affinity peptide based precipitation, and immunobeads based precipitation, having different stringency and yield. All three methods already proved to be efficient in pulling down vesicles from wide range of samples, in particular from complex and viscous samples such as urine, serum, saliva [16]. Although all three methods enabled recovery of EV-RNA from UF and B, we have elected to use CP as it displayed the highest yield and, differently from IA and PA, does not introduce any selective bias in EVs recovery. This feature is considered a plus in the early phase of biomarker and sample characterisation while affinity isolation based methods are the preferential choice for in line coupling with most of the analytical methods relevant for later phase diagnostic developments.

We have also optimised two early steps in sample processing prior the actual EV isolation that can significantly impact overall EV material recovery. The first issue regards the sample preservation conditions that would allow the best sample logistics. Indeed, one of the drawbacks of the current molecular tests for endometrial assessment, that are based on endometrial biopsies, is the need to keep and transport the sample frozen (at −20 °C). This introduces some difficulties in running multicenter projects and increase costs and hurdles in providing centralized diagnostic services. Driven by our prior work and observations on stability of EVs in different matrices (unpublished work) such as blood or urine, we have attempted and compared the long term storage (30 days) of row UF samples, along with serum and urine samples from the same donors, at RT after addition of a commercial preservative. Such storage at RT ensured excellent and even improved stability of EVs in terms of quantity and RNA content in all samples with respect to standard freezing. This friendly-to-routine feature of the sample prompts its transfer to clinical settings and its adoption as the sample of interest in large biomarker trials. Second novel feature of our protocol is introduction of the mucolitic and enzymatic digestion step of the sample as to ensure recovery of vesicles that are bound to and entrapped in mucus. We have adjusted the protocols historically used for recovering the intact cells from mucous samples to the needs of vesicles isolation. This step was in particular critical for the assessment of a brand new source of EVs: cervical cytobrush. The cytobrash has an appeal of being a routine and minimally invasive endocervical sampling technique in gynecological practice. Notably, the presence of exfoliated or abraded endometrial cells in cervical smears is controversial [28,29]. However, our sample processing protocol excludes cells and large debris while providing a very consistent vesicles population from B samples.

UF obtained during the proliferative and secretory phase of the cycle is likely to contain vesicles that come from both major populations of endometrial lining: glandular epithelium and surrounding stromal cells. Due to the fine hormonal regulation, accounting for the cyclic modifications affecting endometrial physiology, we have to expect and consider that the composition of the vesicles collected by UF will reflect the cell type prevalence and condition and will thus significantly change along the cycle providing still unlocked information of potential high diagnostic relevance for monitoring endometrium status in both physiological and pathological settings.

Endometrial epithelium lining and glands proceed from the uterus into the cervical region. Two tissues therefore are in intimate anatomical and functional cooperation in human reproduction, as well as in important pathologies, exemplified by common origin of uterus and cervical cancers. The cell origin composition of the vesicle content of the cervical brush is expected to be more complex than that obtained by uterine flushings, comprising endocervical glandular cells derived vesicles and also those derived potentially from the endometrial cells, squamous cells of exocervical lining, some immune and inflammatory cells, red blood cells, etc.

The quantity and quality, in terms of overall protein content and viscosity of the mucus, itself is changing cyclically in response to estrogen and progesterone stimulation. Particularly in the luteal phase the viscosity, and protein as well as cell load of the mucus would normally increase [30,31]. This complex and changing composition of the mucus renders it a challenging but potentially very informative “multi-tasking” sample: it could also contain surrogate biomarkers reflecting the changes at the endometrium level.

High vesicles content in UF and B with respect to serum and urine increases their appeal as sources for sampling, in particular when additional challenging features of latter samples are considered. Serum contains a very complex mix of vesicles due to contribution of various tissues and in particular blood resident cells creating thus a high background for plausibly very rare endometrium originated exosomes. Urine instead is also collecting contributions from diverse tissues and concentration of particular vesicles is likely to be very low, dependent on very variable urine concentration. Not only is UF very rich in vesicles in terms of absolute numbers but it is also the biofluid in which the vesicle proteins comprise the largest portion of the proteome. It has been proposed that EVs comprise 0.01% of plasma and 0.3% of urine total proteome [32]. Our study indicates that the participation of EVs in the proteome of UF is extremely high, and remains high also in M-UF and B samples with respect to serum and urine samples.

B also features the most abundant source of total RNA, a good portion of which is protected from RNase digestion and therefore likely to be EV encapsulated. Though more impoverished, L-UF also contains consistent amounts of EV associated RNA that is suitable for amplification of EV sorted mRNAs. We are due to notice that our sample processing flow accommodated several stop-and-go steps, where the protocol could be interrupted and samples stored at -20C till the analysis or purification. This complies with the common lab practice and is supported by the observations made on different EV containing biofluids, that the EVS, in particular exosome sized EVs that are the focus of this study, are very resistant to freeze-and-thaw cycles in terms of general structure, number, and immunoreactivity [16]. However, the prominent action of RNAse treatment is likely to be attributed also to a compromised membrane integrity that however does not preclude the recovery of EV encapsulated material.

In this study we have focused on EV contained transcripts as our readout of interest, rather than other RNA species that are found to be more abundant in EVs, such as miRNAs [24,33]. This choice is driven by our interest in identification of tissue specific gene expression profiles that reside in transcriptome of the vesicles as a mirror of tissue gene regulation. Indeed, beyond quantification of paradigmatic mRNAs, namely *GAPDH* and *RNY4*, later shown to be enriched in EVs, we have also addressed the presence of typical endometrial transcripts, including *PAEP*, *ESR1*, and *PGR* mRNA. In particular, glycodeline is a *bona fide* endometrial protein, expressed in a cycle-dependent manner in epithelial cells [34]. Hormone receptors are expressed on both endometrial stromal cells and along the epithelial lining of uterus and cervix. We have detected the endometrial transcripts in both UF and B samples, with the surprising abundance of signal in M-UF that is otherwise poor in total RNA as well as protein content. This finding could indicate the specific cell origin of M-UF and its contained EVs, likely to come from epithelial cells.

Cell heterogeneity in the endometrium is not static phenomena—the number and ratio of two dominant cell types in endometrium varies dependent on the menstrual cycle phase [35]. Therefore, the overall changes in gene expression levels in the endometrium are likely to be a consequence of both dynamic alterations in cell composition and in gene expression in each cell type. Unlike tissue biopsy that is subjected to inter- and intra-sample differences, liquid biopsy is likely to capture both types of changes, and is more representative of total endometrium complexity. EVs that co-express the traits of cell-of-origin and the real-time functional markers are an appealing substrate to track both components of variations, these may be due to physiological regulation or to pathological dysregulation. Ethical hurdles prevented longitudinal sampling from the healthy volunteers and precluded availability of matched biopsy samples for thorough comparison, so we base our evaluation of accordance between surrogate “liquid” biopsy on our previously published data on gene expression in non-pathological endometrium [34]. Briefly, endometrial *PAEP* expression is increased in the early secretory phase (early and mid-luteal phase, days 1to 7 after LH surge) in order to drop later on (LH + 9 and later). Instead this reduction in endometrial *PAEP* is associated with the elevated estrogen receptor, *ESR1*, expression in mid-luteal phase while progesterone receptor *PGR* is downregulated along proceeding of luteal phase favoring the endometrial receptivity for eventual embryo implantation.

Overall, the expression profile for endometrial genes “captured” from UF and B EVs recapitulates well what was expected. In particular, though typically lower, the expression of *PAEP* in B sample mimics the decrease of the expression from early to late secretory phase [34]. *PAEP*, *ESR1*, and *PGR* are indeed normally finely regulated, and their abnormalities in patients with luteal phase defects are associated with infertility [34,36,37].

## 5. Conclusions and Limitations of the Study

Present work describes the set-up of pre-analytical steps, as well as identification of tissue-specific traits, useful for reliable recovery and identification of endometrium originated vesicles in biological samples from uterine flashings and a cervical brush. Considering that the preanalytical variability is the major source of diagnostic uncertainty, providing SOPs for sample storage and processing is fundamental to enable proper biomarker discovery and validation studies and subsequent diagnostics development. Identification of proper markers identifying vesicles coming from the target tissue (endometrium) constitutes the prerogative for full leveraging of advantages of EVs as biomarkers in physiology and pathology conditions. Therefore, despite using a bulk EV isolation method as the initial approach, handy and suitable for routine lab procedures, our work ultimately aims at development of endometrial EV enrichment protocols. Moreover, assessment of a cervical brush as a potential source of endometrial vesicles promises to prompt fast adoption as this sampling procedure is widely used in gynecological monitoring today.

Clear limitations of the present study reside in the sample size, and inability to collect longitudinal samples from healthy and fertile subjects. For the same reason that endometrial biopsies are not performed on healthy donors, direct comparison between tissue and biofluid samples was not performed in this study; nevertheless, we could rely on our prior published work and other available data on expression profiles in healthy endometrium. Therefore, the observations on the quantitative EV features from different sample types, especially the first hints of EV RNA expression profiles along the menstrual cycle, warrant further study. At the same time, this study provides pilot data that justifies sample collection in view of a comparative study between control/fertile individuals and patients undergoing ART procedure in our center. Another limitation correlated to possible ethical hurdles and low compliance of donors or patients, is the fact that, though less invasive than endometrial biopsy, uterine flushing still imposes the need for complex and in-clinic performed procedure, and can cause discomfort for the patient. Therefore, such sampling is easily envisaged as implemented into diagnostic flow of patients undergoing ART while it is not easily adoptable in the scenario of routine screening for common endometrium related conditions and disorders. To this purpose we envisage more work done on brush and urine, while proceeding along the development of methods for selective enrichment of endometrium-originated vesicles from these complex samples. In conclusion, we consider this study a proof of concept, and intend to leverage hereby optimized protocols for further assessment of tissue vs liquid biopsy profiles for preselected gene panels in conditions such as impaired fertility in the case of recurrent implantation failure. Hereby described protocols are currently also being implemented in addressing other endometrium related pathologies such as endometriosis, paving the way to novel solutions in timely diagnostics and accurate management of these perceiving societal challenges.

## Supplementary Materials

The following are available online at https://www.mdpi.com/2073-4409/8/8/811/s1, Figure S1: The overall comparative evaluation of three isolation protocols used for a set-up; Figure S2: Relative EV content in L-UF, M-UF and B across the cycle phases; Table S1: % of recovery at RT preservation vs frozen.
