# Supplementary material for: Clues to Non-Invasive Implantation Window Monitoring: Isolation and Characterisation of Endometrial Exosomes
Source: Cells. 2019 Aug 1;8(8):811. doi: 10.3390/cells8080811 (PMC6721457; doi:10.3390/cells8080811)
Supplement: Supplementary file 1 [file cells-08-00811-s001.zip › Supplementary table 1.docx]

**Supplementary table 1**

|  | % of **recovery at RT preservation vs frozen** | |  |
| --- | --- | --- | --- |
| **Sample types** | **particle concentration/ ml (NTA)** | **EV protein signal intensity (ELISA)** | **Particle size mode (nm. NTA)** |
| **UF** | 84.39 | 106.65 | 103.7 vs 105.8 |
| **S** | 123.42 | 58.97 | 60.9 vs 52.45 |
| **U** | 150.96 | 180.43 | 101.7 vs 101.1 |
