# Supplementary figures and images for: Clues to Non-Invasive Implantation Window Monitoring: Isolation and Characterisation of Endometrial Exosomes
Source: Cells. 2019 Aug 1;8(8):811. doi: 10.3390/cells8080811 (PMC6721457; doi:10.3390/cells8080811)

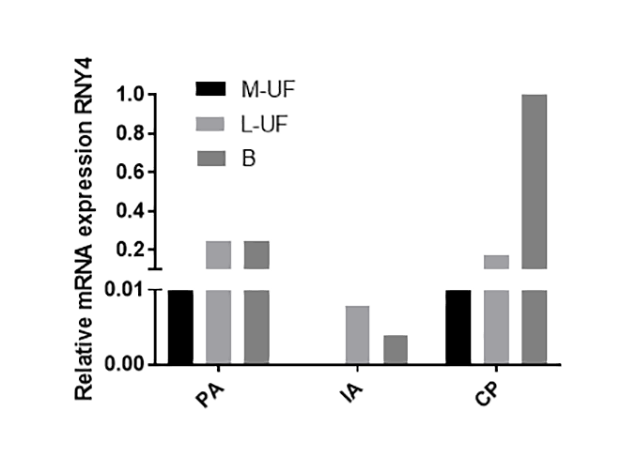

Supplement: Supplementary file 1 [file cells-08-00811-s001.zip › Supplementary Figure 1.tif]

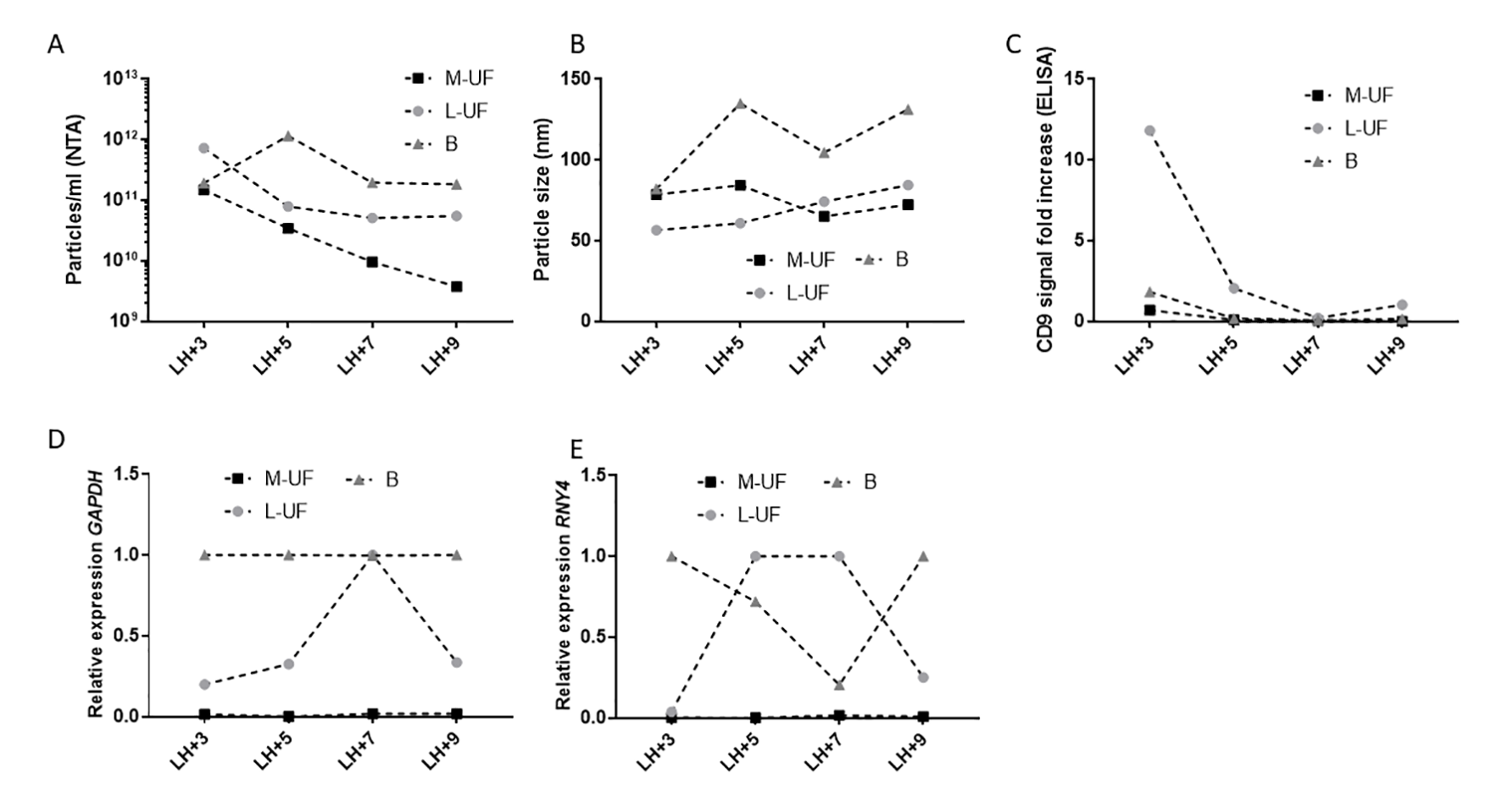

Supplement: Supplementary file 1 [file cells-08-00811-s001.zip › Supplementary Figure 2.tif]
